# Luteolin, a natural flavonoid, inhibits methylglyoxal induced apoptosis via the mTOR/4E-BP1 signaling pathway

**DOI:** 10.1038/s41598-017-08204-6

**Published:** 2017-08-11

**Authors:** Yi Liu, Jie Huang, Xian Zheng, Xia Yang, Yan Ding, Tongyong Fang, Yuyun Zhang, Shuaishuai Wang, Xiaofei Zhang, Xuan Luo, Anlei Guo, Kelly A. Newell, Yinghua Yu, Xu-Feng Huang

**Affiliations:** 1 Jiangsu Key Laboratory of New Drug Research and Clinical Pharmacy, Xuzhou Medical University, Xuzhou, Jiangsu Province China; 2 Jiangsu Key Laboratory of Immunity and Metabolism, Department of Pathogen Biology and Immunology, Xuzhou Medical University, Xuzhou, 221004 China; 3 Illawarra Health and Medical Research Institute, Faculty of Science, Medicine and Health, University of Wollongong, NSW, 2522 Australia

## Abstract

Methylglyoxal (MG) accumulation has been observed in human cerebrospinal fluid and body tissues under hyperglycaemic conditions. Recent research has demonstrated that MG-induces neuronal cell apoptosis, which promotes the development of diabetic encephalopathy. Our previous animal study has shown that luteolin, a natural flavonoid, attenuates diabetes-associated cognitive dysfunction. To further explore the neuroprotective properties of luteolin, we investigated the inhibitive effect of luteolin on MG-induced apoptosis in PC12 neuronal cells. We found that MG inhibited cell viability in a dose-dependent manner and induced apoptosis in PC12 cells. Pretreatment with Luteolin significantly elevated cell viability, reduced MG-induced apoptosis, inhibited the activation of the mTOR/4E-BP1 signaling pathway, and decreased pro-apoptotic proteins, Bax, Cytochrome C as well as caspase-3. Furthermore, we found that pretreatment with the mTOR inhibitor, rapamycin, significantly reduced the expression of the pro-apoptotic protein Bax. Therefore, these observations unambiguously suggest that the inhibitive effect of Luteolin against MG-induced apoptosis in PC12 cells is associated with inhibition of the mTOR/4E-BP1 signaling pathway.

## Introduction

Patients with long-standing diabetes commonly develop diabetic encephalopathy, which is characterized by cognitive decline^1^, neuronal apoptosis^2, 3^, as well as neurochemical and structural abnormalities in the cortex and hippocampus^4, 5^. Although the pathogenesis of diabetic encephalopathy is complex and not fully understood, methylglyoxal (MG) accumulation has been considered as one of the major contributing causes^6, 7^. MG is a reactive dicarbonyl compound physiologically produced from glycolytic pathway intermediates^8, 9^. The MG concentration is significantly increased in the plasma and hippocampal tissue of the brain in patients with diabetes and is closely related to the development of diabetic complications^7, 10^. MG is able to induce cellular damage, neuronal apoptosis and activation of apoptosis related proteins in the brain^11–13^, which play an important role in the pathogenesis of many neurodegenerative disorders^14^.

Mitochondrial apoptosis pathways play a major role in neuronal apoptosis in diabetes. They integrate death signals through Bcl-2/Bax family members and coordinate caspase activation through the release of Cytochrome C (Cyt C). Bcl-2 regulates the translocation of the pro-apoptotic protein, Bax, from the cytosol to the outer mitochondrial membrane^15^. Bax increases membrane permeability and promotes the release of Cyt C, which binds with procaspase-9, resulting in its cleavage to form activated caspase-9^16, 17^. The activated caspase-9, in turn, cleaves procaspase-3 to its active form, which induces cell apoptosis^18^. In the hippocampus of streptozotocin (STZ)-induced diabetic rats, Bax and caspase-3 mRNA or protein levels are considerably increased and related to impaired cognition as measured by the Morris water maze^3^. Therefore, neuronal apoptosis is likely to account for the concomitant emergence of cognitive impairments in the diabetic status.

Mammalian target of rapamycin (mTOR) is a serine/threonine protein kinase, which is activated by PI3K/AKT and involved in the regulation of cellular apoptosis^19–21^. It regulates both protein synthesis and degradation, longevity and cytoskeletal formation^22, 23^. mTOR activates its downstream effector, eukaryotic initiation factor 4E-binding protein 1 (4E-BP1) and ribosomal protein S6 kinase (S6K, also named p70S6K)^24, 25^, which subsequently leads to translation of pro-apoptotic proteins^26, 27^. Through this pathway, mTOR also phosphorylates and inactivates the anti-apoptotic protein Bcl-2^28^. The mTOR signaling pathway is hyperactive in the hippocampus of STZ-induced diabetic mice, while inhibiting mTOR signaling has been shown to prevent the cognitive deficits associated with this model^29^. Collectively, these studies provide potential mechanistic insight for the role of mTOR/4E-BP1 in neuronal apoptosis.

Luteolin is a natural flavonoid that exists in celery, green pepper leaf and seed, chamomile tea, lonicera and medicinal herbs^30^. Previously, luteolin has shown strong anti-apoptotic^31, 32^, anti-oxidant^33^ and anti-inflammatory activities^34^. Luteolin has been found to possess neuroprotective properties *in vivo* and *in vitro* ^35–37^. In our previous animal study we found that luteolin prevented cognitive decline and neuropathological alterations in the cortex and hippocampus of STZ-induced diabetic rats^38^. However, little information is available on the protective effects of luteolin against diabetes-associated neuronal apoptosis. In the present study, we further examined the neuroprotective action of luteolin. Using PC12 cell cultures, which are an established model for the investigation of nervous system disease^39–41^, we examined the role of pro-apoptotic proteins (Bax, Cyt C and caspase-3) and their related signaling molecules mTOR/4E-BP1 in the prevention of MG-induced neuronal apoptosis, which mimics the diabetics state.

## Results

### Luteolin dose dependently prevents MG-induced decreases in cell viability

**Figure 1:**
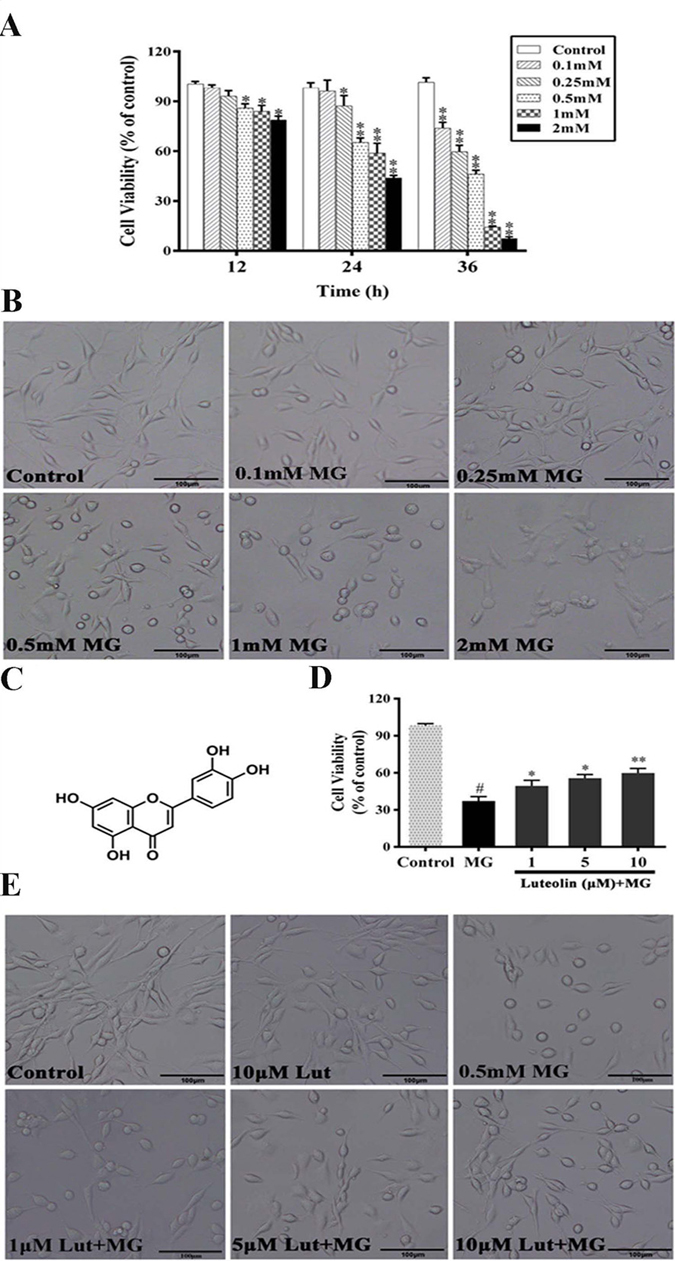
Luteolin (Lut) prevented MG-induced decrease in cell viability in a dose-dependent manner. (**A**) MTT assays were performed to detect viability of PC12 cells treated with MG (0.1–2.0 mM) for 12 h, 24 h and 36 h. Results are expressed relative to control and are presented as means ± SD of three independent experiments, each performed in triplicate. ^*^ *p < *0.05, ^**^ *p* < 0.01 vs control group. (**B**) Representative photographs of cell morphology of PC12 cells treated with MG (0.1–2.0 mM) for 36 h. Morphological changes of PC12 cells were observed by phase-contrasted microscopy. (**C**) Chemical structure of Lut. (**D**) Morphological changes of PC12 cells, pretreated with Lut for 3 h, followed by MG (0.5 mM) for 36 h. Data are presented as means ± SD of three independent experiments, each performed in triplicate. ^#^ *p* < 0.01 vs control group; ^*^ *p* < 0.05 and ^**^ *p* < 0.01 vs MG group. (**E**) Representative images of Lut-induced protection against MG-induced cytotoxicity. Cells were observed by phase-contrast microscopy.

The cytotoxicity of MG was examined by the MTT assay. PC12 cells were treated with various concentrations of MG (0.1, 0.25, 0.5, 1 and 2 mM) and cell viability was examined at 12 h, 24 h and 36 h after MG exposure. Cell viability decreased with increasing concentrations of MG and incubation time (Fig. 1A). MG significantly reduced cell viability after 12 hours of incubation (0.5, 1 and 2 mM), 24 hours of incubation (0.25–2 mM) and 36 hours of incubation (all concentrations) (all *p* < 0.05). Phase-contrast microscopy revealed a significant reduction in the number of cells, a loss of cellular neurites, shrinkage or swelling of cell bodies and disruption of the dendritic networks in PC12 cells exposed to MG (all concentrations) for 36 hours, compared to the control group (Fig. 1B). Collectively, these findings suggest that exposure of PC12 neuronal cells to MG induces cytotoxicity and decreases cell viability.

To determine the effect of luteolin on MG-induced cytotoxicity, PC12 cells were pretreated with 1, 5 and 10 μM of luteolin for 3 h, followed by 0.5 mM MG for 36 h. Luteolin dose-dependently prevented MG-induced reductions in cell viability, as examined by MTT (Fig. 1D). Phase-contrast microscopy images further confirmed the protective effect of luteolin on MG-induced cytotoxicity in PC12 cells (Fig. 1E).

### Luteolin inhibits MG-induced apoptosis

**Figure 2:**
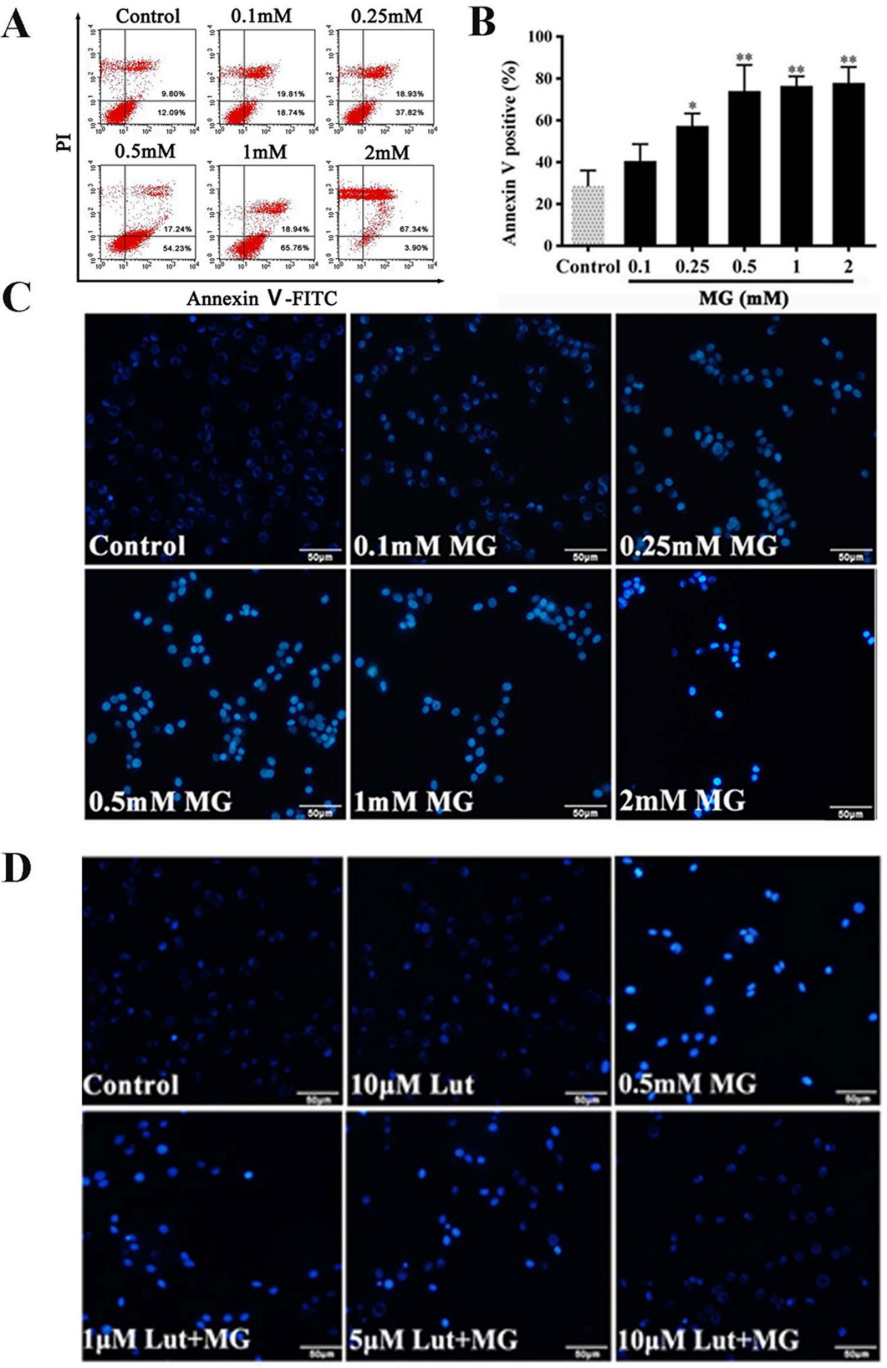
Luteolin (Lut) inhibited MG-induced cell apoptosis. Apoptosis assessment of PC12 cells treated with MG (0.1–2.0 mM) for 36 h. Lut protected PC12 cells against MG-induced apoptosis. (**A**) Cells were stained by fluorescent annexin V and propidium iodide (PI) and then examined for apoptosis by flow cytometry. (**B**) The percent of Annexin V positive cells following increasing concentrations of MG. Data are representatives from three independent experiments and the percentages of different populations were labeled in the figures. ^*^ *p* < 0.05 and ^**^ *p* < 0.01 vs control group. (**C**) Nuclear fragmentation was assessed by nuclei staining with Hoechst 33358. MG (0.1–2 mM) increased the apoptosis in PC12 cells. (**D**) Cells were pretreated with Lut (1, 5, 10 μM) for 3 h, followed by 0.5 mM MG exposure for 36 h. Morphological apoptosis was determined by Hoechst 33258 staining.

Using Annexin V-FITC/PI staining and flow cytometry, we examined whether MG-induced growth inhibition was a result of apoptosis. As shown in Fig. 2A, treatments with 0.1–2 mM MG resulted in an increase of apoptotic cells (both Annexin V-FITC-/PI- and Annexin V-FITC-/PI+). MG, dose dependently, increased rates of apoptosis in PC12 cells, with apoptosis rates of the MG groups (0.25, 0.5, 1, 2 mM) being significantly higher than that of the Control group (Fig. 2B, *p* < 0.05).

Hoechst 33258 staining (Fig. 2C) also revealed that PC12 cells in the MG groups acquired typical features of apoptosis, including cell shrinkage, nuclear pyknosis and apoptotic bodies (Fig. 2C). Luteolin (5 and 10 μM) alleviated MG (0.5 mM) induced morphological changes of apoptosis in PC12 neuronal cells (Fig. 2D).

### Luteolin inhibits the MG-induced activation of mTOR-4E-BP1

mTOR has a role in neurodegenerative diseases. Here we found that the mTOR inhibitor, rapamycin (Rap), inhibited PC12 cell viability at high (10 μM) but not low (0.1 and 1 μM) concentrations (see Supplementary Fig. S1A). Furthermore, Rap prevented the MG-induced reduction in cell viability using an MTT assay (see Supplementary Fig. S1B). Pretreatment of PC12 cells with 0.1 or 1 μM Rap significantly enhanced cell viability compared with the MG-treatment group, returning cell viability to 60% of control levels (*p* < 0.05). Apoptosis was subsequently measured by TUNEL (red) and DAPI (blue) staining after Rap at 1 μM and MG (0.5 mM) incubation (see Supplementary Fig. S1C). The number of TUNEL-positive cells in the MG treated group significantly increased compared with the control group (*p* < 0.05), while the number of TUNEL-positive cells was markedly reduced in the Rap (1 μM) + MG (0.5 mM) treated group compared with MG treated group (*p* < 0.05) (see Supplementary Fig. S1D). Therefore, the inhibition of mTOR by Rap could alleviate MG induced PC12 cell apoptosis, suggesting MG-induced reductions in cell viability and apoptosis could occur, at least in part, via mTOR.

**Figure 3:**
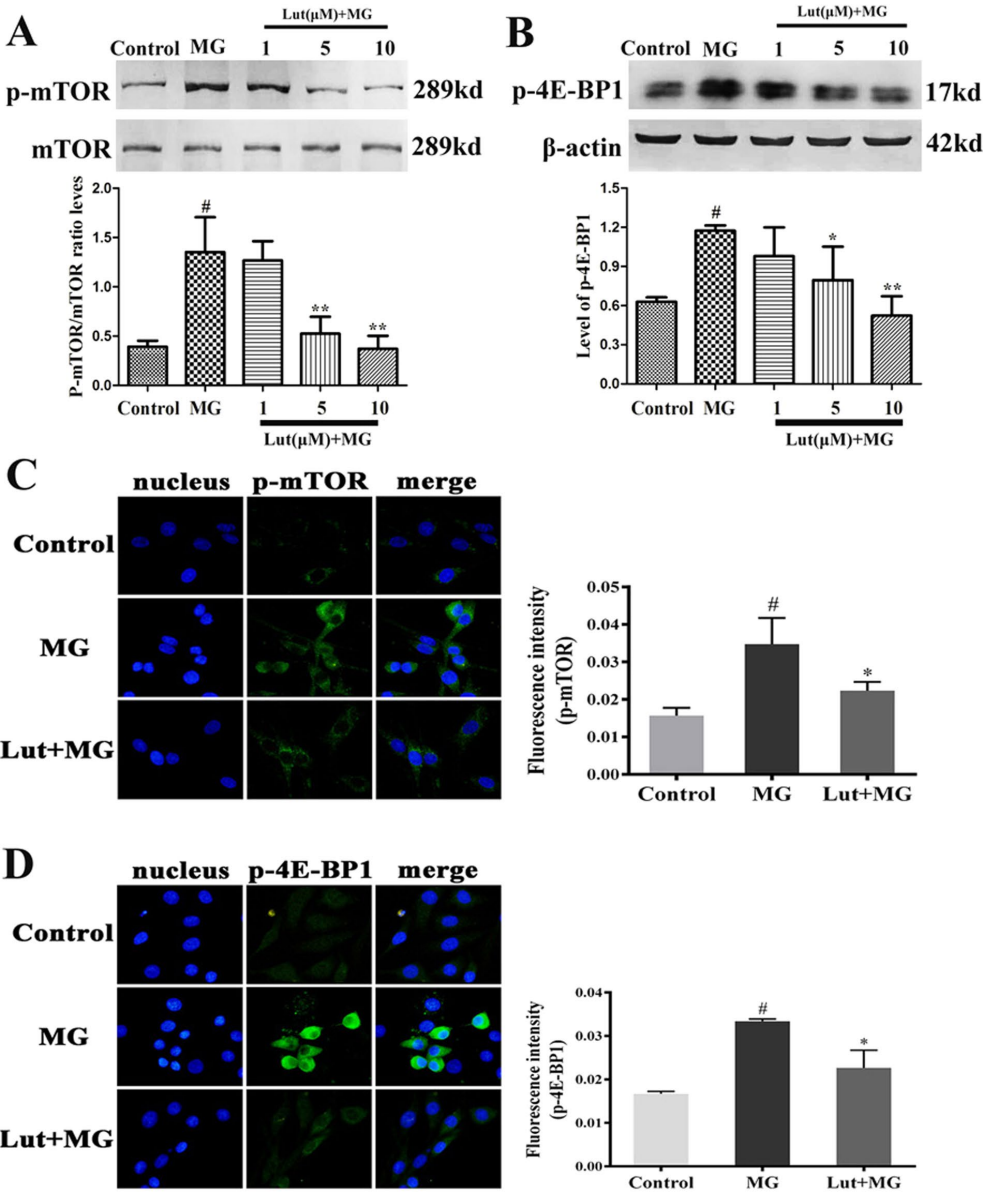
Luteolin (Lut) inhibited the activation of mTOR-4E-BP1 induced by MG. PC12 cells were treated with the Lut (1, 5 and 10 μM) for 3 h, followed by incubating with 0.5 mM MG for 24 h. The activation of mTOR (**A** and **C**) and 4E-BP1 (**B** and **D**) was determined by western blot and immunofluorecence staining. Data are presented as means ± SD of three independent experiments, each performed in triplicate. ^#^ *p* < 0.01 vs control group; ^*^ *p* < 0.05 and ^**^ *p* < 0.01 vs MG group. The full-length blots/gels are presented in Supplementary Figs S4 and S5.

To further determine whether the ability of luteolin to prevent MG-induced apoptosis is via inhibition of mTOR and its downstream effector 4E-BP1, we measured p-mTOR and p-4E-BP1 levels by western blot and immunofluorescence staining. As shown in Fig. 3A, the phosphorylation of mTOR was significantly increased in the MG treated group compared with the control group (*p* < 0.05), while luteolin (5 and 10 μM) decreased the phosphorylation of mTOR compared with MG treated group (*p* < 0.05), returning p-mTOR to control levels. Luteolin (5 and 10 μM) also prevented the MG-induced increase in p-4E-BPI (Fig. 3B). To further confirm the results, we determined the levels of mTOR and 4E-BP1 by immunofluorescence staining. As shown in Fig. 3C and D, the fluorescence intensity of p-mTOR and p-4E-BP1 significantly increased in the MG group, while the intensity of these two were significantly lower in the luteolin 10 μM + MG group compared with MG group. These observations suggest that luteolin potentially inhibits mTOR and may have a similar effect to Rap in PC12 cells.

Furthermore, p-AKT (mTOR upstream molecule) and p-p70S6K (p-4E-BP1 downstream molecule) were examined by Western blot. The phosphorylation of AKT was significantly increased in the MG group compared with Control group (*p* < 0.05), while luteolin pre-treatment (5 and 10 μM) decreased the p-AKT compared with MG group (*p* < 0.05) (see Supplementary Fig. S3A). Luteolin (5 and 10 μM) also prevented the MG-induced p-p70S6K (see Supplementary Fig. S3B).

### Luteolin inhibits MG-induced expression of the apoptosis related proteins, Bax, Cyt C and caspase-3

There is crosstalk between mTOR and Bax, which acts as a gateway for caspase-mediated apoptosis. Compared with the control group, the protein level of Bax was significantly reduced in the Rap (1 μM) treated group as measured by western blot and fluorescence staining (both *p* < 0.05; see Supplementary Fig. S2A and S2B), suggesting that inhibition of mTOR decreased apoptosis markers.

**Figure 4:**
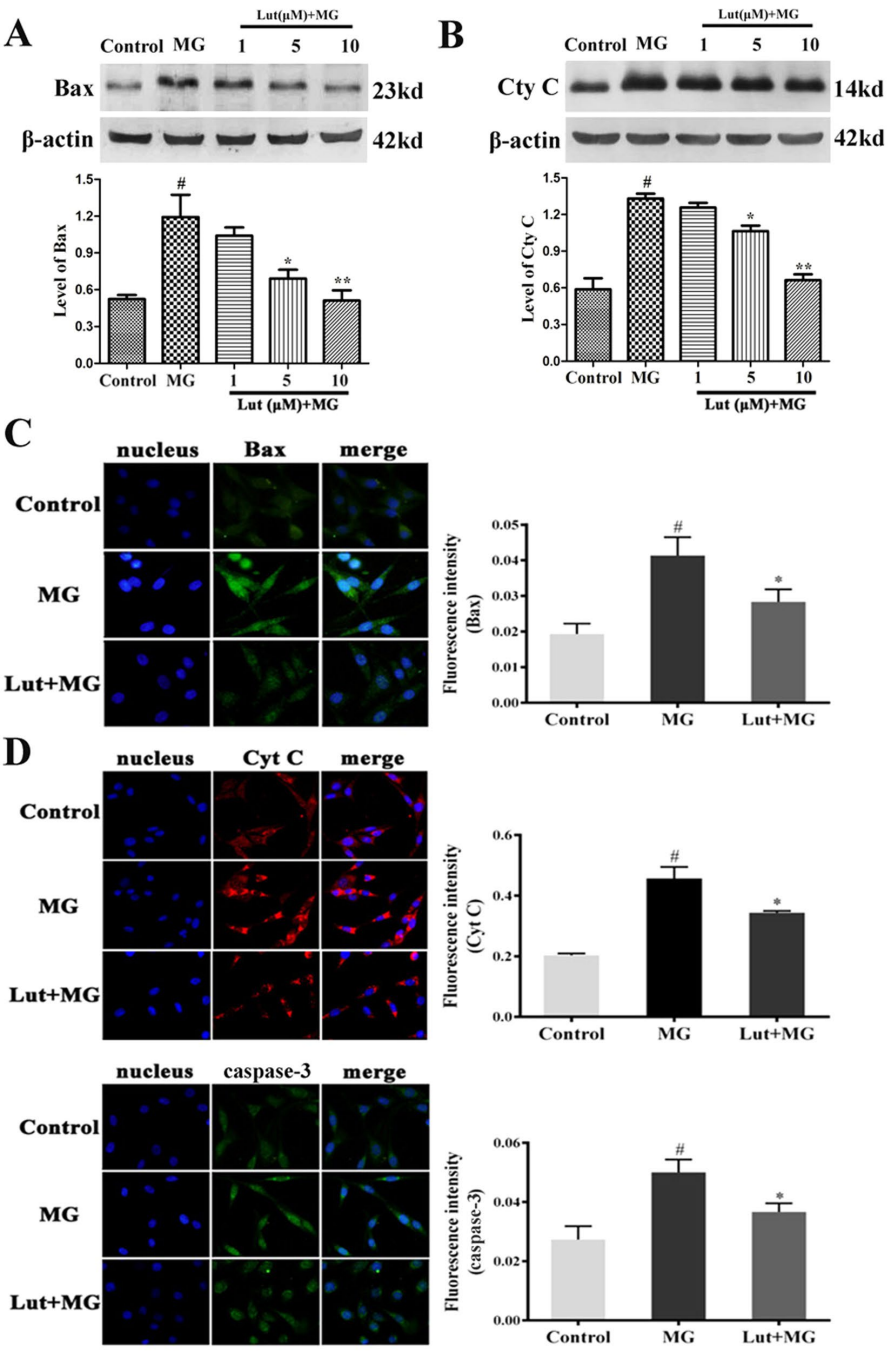
Luteolin (Lut) inhibited MG-induced overexpression of Bax, Cyt C and caspase-3. The cells were pretreated with Lut (1, 5, 10 μM) for 3 h, followed by 0.5 mM MG administration for an additional 36 h. Bax (**A**) and Cyt C (**B**) were determined by western blotting analysis and the band densities were normalized with β-actin. Data are presented as means ± SD of three independent experiments, each performed in triplicate. ^#^ *p* < 0.01 vs control group; ^*^ *p* < 0.05 and ^**^ *p* < 0.01 vs MG group. The expression of Bax (**C**), Cyt C (**D**) and caspase-3 (**E**) was analyzed by immunofluorescent staining. Histograms show the quantification of the fluorescence intensity of the corresponding proteins. ^#^ *p* < 0.01 vs control group; ^*^ *p* < 0.05 vs MG group. The full-length blots/gels are presented in Supplementary Figs S6 and S7.

Our results thus far suggest that luteolin could be a potential mTOR inhibitor, so next we examined if luteolin may affect the apoptosis related proteins, Bax, Cyt C and caspase-3. As shown in Fig. 4A and B, the protein levels of Bax and Cyt C significantly increased in the MG-treated group compared with the control group (all *p* < 0.05). Pretreatment with luteolin (1, 5 and 10 μM) for 3 h dose-dependently decreased the protein levels of Bax and Cyt C compared with the MG-treated group as examined by western blot (Fig. 4A and B). To further confirm the results, we determined the level of Bax, Cyt C and caspase-3 by immunofluorescent staining. As shown in Fig. 4C,D and E, the fluorescence intensity of Bax, Cyt C and caspase-3 were significantly increased in the MG-treated group compared with the control group (all *p* < 0.05). Pretreatment with 10 μM luteolin for 3 h significantly decreased the fluorescence intensity of Bax, Cyt C and caspase-3 compared with the MG group (all *p* < 0.05).

## Discussion

Diabetic encephalopathy is now an accepted complication of diabetes and has become the focus of research in this field^42, 43^. High levels of MG have been found in the plasma of diabetic individuals^44^. The neurotoxicity of MG plays a causal role in the development of diabetic encephalopathy^7^. Our results confirm that MG inhibited cell viability in a dose-dependent manner and induced apoptosis of PC12 neuronal cells as measured by MTT, Hoechst 33258 dye staining and Annexin V-FITC/PI dual staining. These results were consistent with previous findings^45^, collectively highlighting that apoptosis is a major manner by which MG induces neuronal progenitor death. Our study extended this further to show that MG-induced apoptosis occurred via activation of mTOR signaling and the pro-apoptosis related Bax protein. More interestingly, we also found that the natural flavonoid, luteolin, could protect against MG induced apoptosis.

We have previously shown that luteolin protected against high fat diet-induced cognitive deficits in obese pre-diabetic mice^46^ and in STZ-induced diabetic rats^38^. In the present study, we demonstrated that luteolin prevented MG-induced neuronal apoptosis, evidenced by the 37–60% increase in cell viability (measured by MTT) after pretreatment with luteolin (1–10 μM). Furthermore, the rate of MG-induced apoptotic PC12 cells was reduced after luteolin pretreatment, as detected by Hoechst 33258 dye staining. These findings suggest that luteolin affords protection against MG-induced neuronal apoptosis. Combined with our previous animal studies^38, 46^, the present cell study suggests that the ability of luteolin to prevent apoptosis may contribute to its ability to improve cognitive deficits in diabetes.

The mitochondrial apoptosis pathway, Bcl-2/Bax/Cyt C/caspase-3, plays a major role in neuronal apoptosis in diabetes^47–49^. In the present study, luteolin prevented MG-induced activation of the pro-apoptotic Bax protein in PC12 cells, whilst also suppressing Cyt C and caspase-3 levels. Therefore, luteolin-induced reductions of the mitochondrial apoptosis pathway may inhibit neuronal apoptosis, contributing to an improvement of cognition in diabetes patients with elevated levels of MG.

Experimental evidence suggests that several pathways mediate AKT/mTOR-induced apoptosis, and one of these involves the Bax protein^27^. Bax, a pro-apoptotic member of the Bcl-2 family of proteins, is a target of mTOR^50, 51^. In cancer cells it has been shown that mTOR activates its downstream effector 4E-BP1 and p70S6K, which subsequently leads to translation of pro-apoptotic proteins^27, 52^ as well as phosphorylates and inactivation of the anti-apoptotic protein Bcl-2^28^. Consistent with these findings, our study confirmed that the mTOR/4E-BP1 signaling pathway modulates apoptosis and regulates the expression of the pro-apoptotic protein Bax in the PC12 cell. We report that Rap, as a blocker of mTOR/4E-BP1 signaling, significantly increased cell viability and decreased TUNEL-positive cell numbers, suggesting that inhibition of the mTOR/4E-BP1 signaling pathway protected PC12 cells from MG-induced neuronal apoptosis. In addition, immunofluorescent staining and western blotting analysis indicated that Rap pretreatment significantly inhibited the expression of the pro-apoptotic protein Bax. Previously, it was found that phosphorylated mTOR was significantly increased in the hippocampus of STZ-induced diabetic mice, while inhibiting mTOR signaling by Rap prevented the cognitive deficits in this model^29^. Therefore, these findings including ours indicate that blocking mTOR/4E-BP1 down-regulates the expression of the pro-apoptotic protein Bax which may be important for reducing neuronal apoptosis during high MG status.

**Figure 5:**
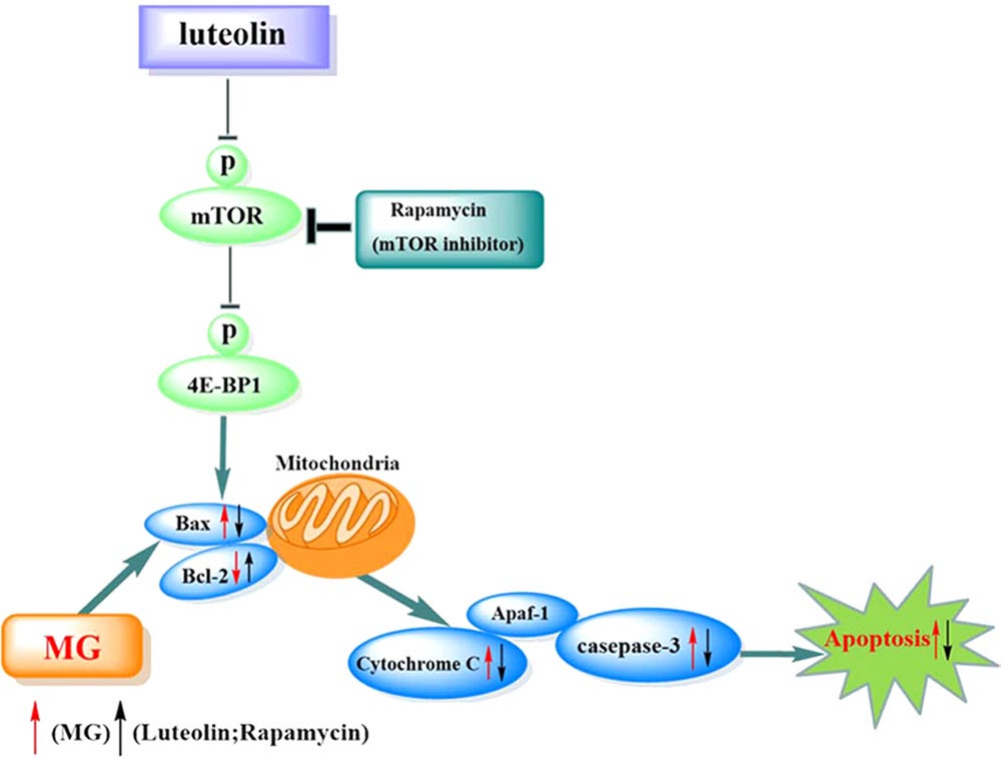
A proposed model of molecular targets of Luteolin (Lut) in preventing MG-induced apoptosis. Our study found that Lut prevents MG-induced apoptosis by decreasing protein phosphorylation of mTOR, and 4E-BP1 in PC12 cells. Furthermore, our study also confirmed the mTOR/4E-BP1 signaling pathway modulates apoptosis and regulates the expression of the pro-apoptotic protein Bax in PC12 cells. This suggests that Lut prevented MG-induced cell apoptosis and decreased the expression of the pro-apoptotic Bax protein and Cyt C and casepase-3 through inhibiting mTOR/4E-BP1 signaling pathway.

Interestingly, we found that luteolin pretreatment significantly inhibited the MG-induced activation of the mTOR/4E-BP1 signaling pathway in PC12 neuronal cells. This suggests that luteolin may act as an mTOR inhibitor to reduce neuronal apoptosis. mTOR plays a key role in diabetes and its related Alzheimer’s pathogenesis^29, 53^, with an upregulation of mTOR reported in the brains of STZ-induced diabetic rodents^29, 54^ as well as in AD patients^55^. Altered mTOR signaling in AD was subsequently found to be associated with cognitive decline^56^. Modulating mTOR activity therefore provides an attractive avenue to discover new therapies to attenuate diabetic-related cognitive decline and prevent diabetic encephalopathy and AD. Here, we found that luteolin is a potent inhibitor similar as rapamycin to block mTOR/4E-BP1 as well as the expression of AKT and p70S6K and down-regulate the expression of pro-apoptotic protein Bax, Cyt C and casepase-3, indicating that it could be developed into an effective treatment for cognitive decline (Fig. 5). While this has not been trialed in diabetes or AD patients, two pilot, open-label, clinical studies using a luteolin-containing dietary formulation reported significant improvement in attention and sociability in children with Autism^57, 58^, supporting the potential transition of luteolin to the clinic for the treatment of diabetic encephalopathy and AD.

In summary, the findings presented in this study increase our understanding of the mechanistic pathway by which mTOR-induced neuronal apoptosis may play an important causal role in the MG-induced pathogenesis of diabetic encephalopathy. Targeting mTOR may provide important novel therapeutic approaches for MG-induced diabetic encephalopathy. Luteolin, a herb derived natural flavonoid, improved MG-induced cell apoptosis and decreased the expression of the pro-apoptotic Bax protein as well as Cyt C and casepase-3. Furthermore, luteolin acts as an mTOR inhibitor contributing to protection against MG-induced neuronal apoptosis. In addition, flavonoid luteolin could target multiple signaling kinases, and not necessarily only the mTOR and apoptotic pathways, for its neuroprotective effects. Further research into the effects of luteolin on signaling pathways involved in neuroinflamamtion and oxidative stress should be investigated as flavonoids are considered to be capable of counteracting neuroinflammation and oxidative stress.

## Materials and Methods

### Materials

3-(4,5-Dimethylthizol-2-yl)-2,5-diphenyltetrazolium bromide (MTT) and MG (40%, w/v), Propidium Iodide (PI) were purchased from Sigma-Aldrich (St. Louis, MO, USA). Luteolin (purity > 98%) was purchased from Shanxi Sciphar Biotechnology Co., Ltd (Shanxi, China). Fetal bovine serum (FBS) and Dulbecco’s modified Eagle medium (DMEM) were obtained from Gibco (Grand Island, NY, USA). mTOR, phospho-mTOR (Ser2448), phospho-4E-BP1 (Thr37/46), AKT, phospho-AKT (Ser473), phospho-p70S6K (Thr389) were purchased from Cell Signaling Technology (Danvers, MA, USA). Anti-Cytochrome C antibody was purchased from Abcam (Cambridge, UK). Antibodies against Bax and Bcl-2 were obtained from Santa Cruz Biotechnology (Santa Cruz, CA, USA). Mouse anti-β-actin monoclonal antibody was purchased from Abmart (Shanghai, China). All secondary antibodies were obtained from Santa Cruz Biotechnology (Santa Cruz, CA, USA).

### Cell culture and treatment

PC12 cells were obtained from the Chinese Type Culture Collection (Shanghai, China). The cells were grown in DMEM containing 10% FBS, 100 U/mL penicillin and 100 U/mL streptomycin in a humidified atmosphere of 95% air and 5% CO_2_ at 37 °C. For experiments indicated below, PC12 cells were exposed to MG at various concentrations (0.1–2 mM) for different time periods (12, 24, 36 h). Luteolin (1, 5, 10 μM) was added 3 h prior to MG administration. Rapamycin (Rap) (1 μM) was added 1 h prior to MG administration.

### Cell viability assay

PC12 cells were seeded in 96-well plates at a density of 5 × 10^3^ cells/well. The cells were grown for 12 h, and the medium was changed to that containing various concentrations (0.1, 0.25, 0.5, 1 and 2 mM) of MG. All measurements were performed at 36 h after the cells were exposed to MG. Cytotoxicity of MG was measured by MTT assay.

PC12 cells were pretreated with different concentrations (1, 5, 10 μM) of Luteolin, after which they were cultured with 0.5 mM MG for 36 h. MTT (0.5 mg/mL) was then added to each well and the cells were cultured for an additional 4 h. Finally, the MTT was carefully removed by aspiration, the formazan crystals were dissolved in dimethyl sulfoxide and the absorbance was read at 550 nm using a microplate reader.

### Observation of morphologic changes

PC12 cells were seeded in 6-well plates at a density of 7 × 10^4^ cells/well. The cells were grown for 12 h, and the medium was changed to that containing various concentrations (0.1, 0.25, 0.5, 1 and 2 mM) of MG. After the cells were exposed to MG for 36 h, the cellular morphology was observed under a phase-contrast microscope at 100x magnification with a CCD camera (OLYMPUS IX73, Japan).

### Nuclear staining with Hoechst 33258

To assess changes in nuclear morphology during apoptosis, cells were stained with the fluorescent nuclear dye Hoechst 33258. Briefly, PC12 cells were seeded into 6-well plates at a density of 7 × 10^4^ cells/well and incubated at 37 °C for 12 h with various concentrations of different experimental compounds. After treatment, cells were fixed with paraformaldehyde (4.0%), washed twice with ice-cold PBS and stained with Hoechst 33258 staining solution for 10 min at room temperature. Using inverted fluorescent microscopy, fragmented or condensed nuclei were scored as apoptotic based on their morphology.

### TUNEL assay

Terminal deoxynucleotidyl transferase dUTP nick end labeling (TUNEL) is a method for detecting DNA fragmentation by labeling the terminal end of nucleic acids. Apoptosis was evaluated with the TUNEL BrightRed Apoptosis Detection kit (Vazyme Biotech Co., Ltd. Nanjing, China) according to the manufacturer’s instructions. Briefly, 5 × 10^4^ cells/ml were plated in 6-well flat-bottom plates and pretreated with 1 μM Rap for 1 h and then treated with 0.5 mM MG for 36 h. Cells were fixed in 4% paraformaldehyde at 4 °C for 30 min, and then permeabilized in 0.1% Triton X-100. Cells were then washed and stained with TUNEL reaction mixture and DAPI (Sigma, St. Louis, MO). The image was visualized and captured by microscope (Olympus × 51 W, Olympus Microsystems).

### Flow cytometry analysis for apoptosis

The apoptotic cells were quantitated using annexin V-FITC/Propidium Iodide (PI) apoptosis assay kits. Briefly, PC12 cells were seeded into 6-well plates at a density of 1 × 10^5^ cells/well and incubated with various concentrations of different experimental compounds at 37 °C for 12 h. After treatment, PC12 cells were washed twice with PBS and detached by pipette. The cells were then centrifuged at 1000 rpm for 5 min and washed with PBS twice. Cells were stained with Annexin V-FITC and PI by using Annexin V-FITC kit (Jiangsu KeyGEN BioTECH Co., Ltd. Jiangsu, China) and collected for flow cytometry analysis with emission filters of 525 and 575 nm, respectively. Approximately 1 × 10^4^ counts were made for each sample. The percentages of early apoptotic (Annexin V-FITC^+^/PI^−^) and late apoptotic (Annexin V-FITC^+^/PI^+^) cells were calculated by CELL Quest software.

### Immunofluorescent staining

PC12 cells were seeded on glass cover slips in a 24-well plate at 1 × 10^4^ cells/well and cultured with various concentrations of different experimental compounds at 37 °C for 12 h. After treatment, PC12 cells were washed three times with ice-cold PBS, immediately fixed in 4% paraformaldehyde for 30 min and permeabilized with 0.5% Triton X-100 for 15 min. The cells were incubated with primary antibodies against Bax, caspase-3, p-mTOR and p-4E-BP1 (1:50 dilution) overnight at 4 °C. Cells were then washed three times with PBS, incubated with FITC-conjugated goat anti-rabbit secondary antibody (1:200 dilution) for 2 h at room temperature. Cells were washed three times in PBS, and stained with DAPI or PI (10 μg/mL) for nuclear identification. The image was visualized and captured by microscope (Olympus x51 W, Olympus Microsystems).

### Western blot analysis

After treatment as described above, PC12 cells were washed twice with ice-cold PBS (pH 7.4) and centrifuged at 1000 rpm for 5 min. Cell pellets were lysed in an ice cold extraction buffer (20 mM Tris-HCl buffer, pH 7.6, 150 mM NaCl, 2 mM EDTA·2Na, 50 mM sodium fluoride, 1 mM sodium vanadate, 1% Nonidet^TM^ P-40, 1% sodium deoxycholate, 0.1% SDS, 1 mg/ml aprotinin, and 1 mg/ml leupeptin). Cell lysates were centrifuged at 12000 g for 15 min at 4 °C. The supernatant was collected and used for further analysis.

The protein concentration was determined by the BCA Protein Assay Kit (Sigma–Aldrich) using bovine serum albumin (BSA) as the standard. Equal amounts of protein (50 μg protein/lane) were electrophoresed on 8–12% density SDS-acrylamide gels. Following electrophoresis, the proteins were transferred to a nitrocellulose filter (NC) membrane using an electric transfer system. The membrane was blocked with 5% (v/v) skim milk powder in Tris-buffered saline with Tween 20 (TBST; 10 mM Tris-HCl, 150 mM NaCl and 0.1% Tween 20, pH 7.5) at room temperature for 1 h. The membranes were incubated with primary antibodies against Bcl-2 (1:600), Bax (1:500), Cytochrome C (1:600), mTOR (1:600), phospho-mTOR (1:500), AKT (1:100), phospho-AKT (1:1000), phospho-4E-BP1(1:1000), phospho-p70S6K (1:1000) and β-actin (1:500) overnight at 4 °C. The membranes were washed three times with 1 × TBST for 5 min each. The membranes were incubated with the appropriate horseradish peroxidase (HRP)-conjugated secondary antibody at room temperature for another 2 h and washed again three times in TBST buffer.

### Statistical analysis

Data were expressed as mean ± SD of at least three independent experiments. Data were analyzed by one-way ANOVA followed by Dunnett’s post hoc test. *p* < 0.05 was considered to be statistically significant. Statistical analysis was conducted using SPSS 16.0 (SPSS Inc., Chicago, IL, USA).

## Electronic supplementary material

supplement information
