# Supplementary material for: Luteolin, a natural flavonoid, inhibits methylglyoxal induced apoptosis via the mTOR/4E-BP1 signaling pathway
Source: Sci Rep. 2017 Aug 11;7:7877. doi: 10.1038/s41598-017-08204-6 (PMC5554232; doi:10.1038/s41598-017-08204-6)
Supplement: Supplementary file 1 — supplement information [file 41598_2017_8204_MOESM1_ESM.doc]

**Supplementary Information**

**Luteolin, a natural flavonoid, inhibits methylglyoxal induced apoptosis via the mTOR/4E-BP1 signaling pathway**

Yi Liu^1, *^, Jie Huang^1^, Xian Zheng^1^, Xia Yang^1^, Yan Ding^1^, Tongyong Fang^1^, Yuyun Zhang^1^, Shuaishuai Wang^1^, Xiaofei Zhang^1^, Xuan Luo^1^, Anlei Guo^1^, Kelly A. Newell^3^, Yinghua Yu^2, 3 *^, Xu-Feng Huang^2, 3^

^1^ Jiangsu Key Laboratory of New Drug Research and Clinical Pharmacy, Xuzhou Medical University, Xuzhou, Jiangsu Province, China

^2^ Jiangsu Key Laboratory of Immunity and Metabolism, Department of Pathogen Biology and Immunology, Xuzhou Medical University, Xuzhou 221004, China

^3^ Illawarra Health and Medical Research Institute, Faculty of Science, Medicine and Health, University of Wollongong, NSW 2522, Australia

* Corresponding. author:

Yi Liu, E-mail: [cbpeliuyinew@163.com](mailto:cbpeliuyinew@163.com)

Yinghua Yu, E-mail: [yinghua@uow.edu.au](mailto:yinghua@uow.edu.au)


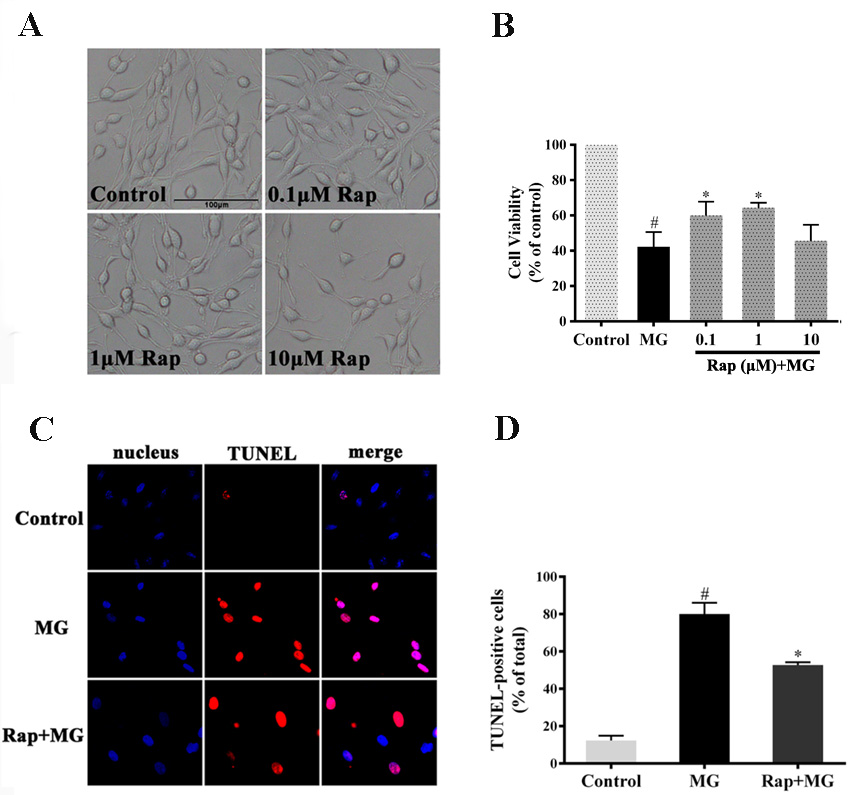


**Supplementary Figure S1. Inhibition of mTOR by rapamycin (Rap) activation promotes the survival of PC12 cells.** (A) Representative photographs of cell morphology after treatment of Rap (0.1, 1, 10 μM) for 1 h. Cells were observed by phase-contrast microscopy. Bar = 100 μm. (B) PC12 cells were incubated with Rap (0.1, 1, 10 μM) for 1 h followed by MG (0.5 mM) treatment for 36 h. ^*^*p* < 0.05 vs control group. (C) Representative microphotographs showed Rap (1 μM) prevented MG (0.5 mM) induced increase in TUNEL. (D) Histograms showing ratios of TUNEL-positive cells as a percent of total cells. ^#^*p* < 0.01 vs control group;^*^*p* < 0.05 vs MG group.


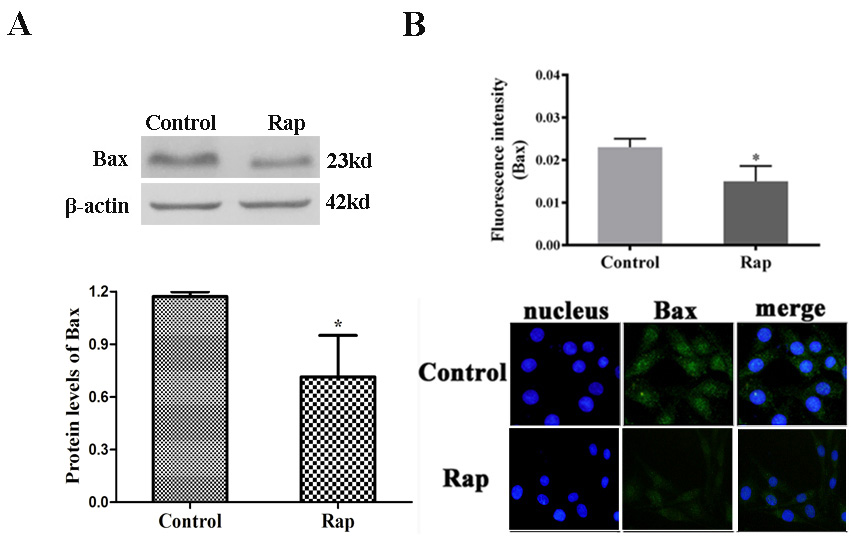


**Supplementary Figure S2. mTOR was required for the expression of Bax protein.** (A) Pretreatment of rapamycin (Rap) reduced the expression of Bax protein in PC12 cells by Western blot. (B) Immunofluorescent staining showed Rap treatment inhibited Bax protein expression. Data are presented as means ± SD of three independent experiments, each performed in triplicate.^*^*p* < 0.05 vs control group. The full-length blots/gels are presented in Supplementary Figure S8.


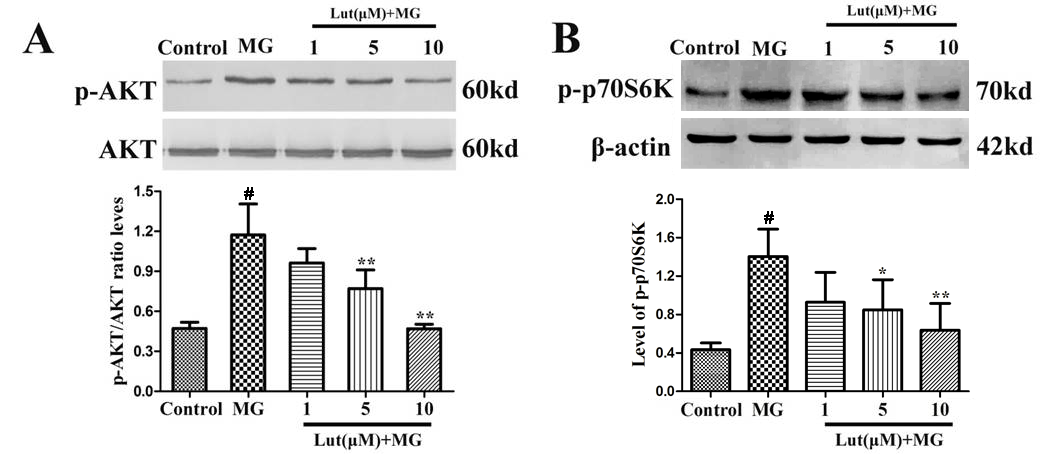


**Supplementary Figure S3. Luteolin (Lut) inhibited the activation of AKT-mTOR-p70S6K induced by MG.** PC12 cells were treated with the Lut (1, 5 and 10 μM) for 3 h, followed by incubating with 0.5 mM MG for 24 h. The activation of AKT (A) and p70S6K (B) was determined by western blot. (A) and (B) Lut inhibited the p-AKT and p-p70S6K induced by MG. Data are presented as means ± SD of three independent experiments, each performed in triplicate. ^#^*p* < 0.01 vs control group; ^*^*p* < 0.05 and ^**^*p* < 0.01 vs MG group. The full-length blots/gels are presented in Supplementary Figure S9 and S10.

p-mTOR (289 kd) mTOR (289 kd)

(Ser2448)

A


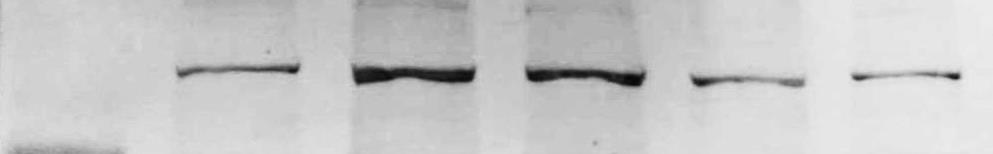

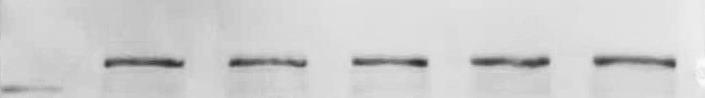


B


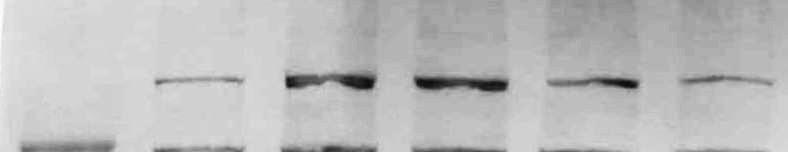

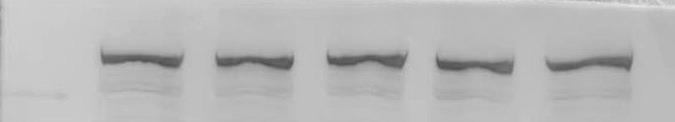


C


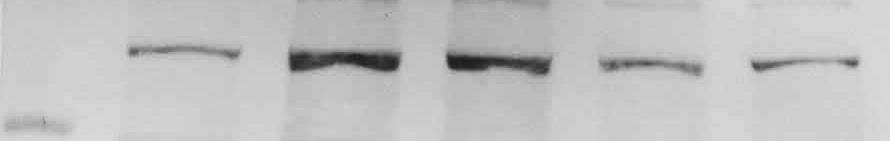

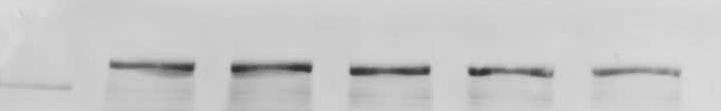


**Supplementary Figure S4.** **The full-length blots/gels images of Figure 3A.**The Figure showed the full-length blots/gels images of p-mTOR and mTOR, in which the Supplementary Figure S4A was the corresponding original uncropped images for Figure 3A in the main manuscript, and other images were parallel test data.

p-4EBP1 (17 kd) β-actin (42 kd)

(Thr37/46)

A


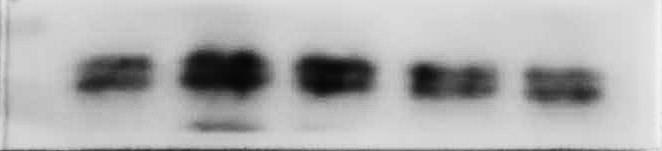

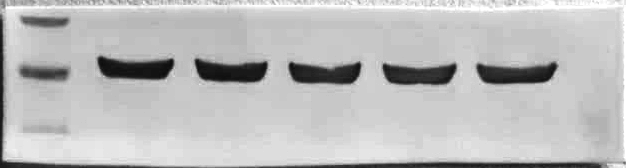


B


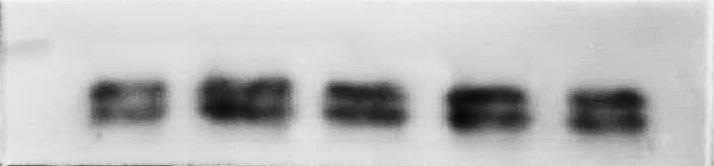

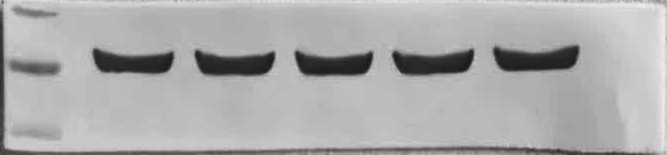


C


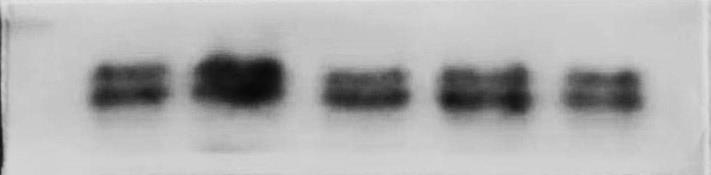

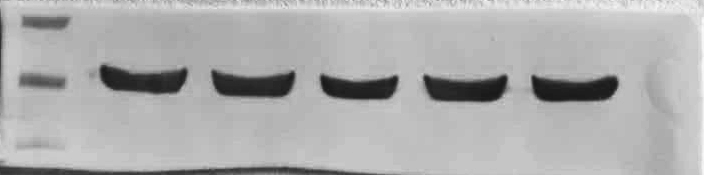


**Supplementary Figure S5.** **The full-length blots/gels images of Figure 3B.**The Figure showed the full-length blots/gels images of p-4EBP1 and β-actin, in which the Supplementary Figure S5A was the corresponding original uncropped images for Figure 3B in the main manuscript, and other images were parallel test data.

Bax (23 kd) β-actin (42 kd)

A


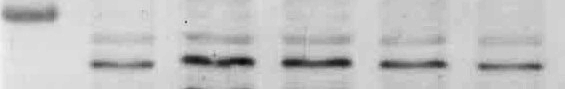

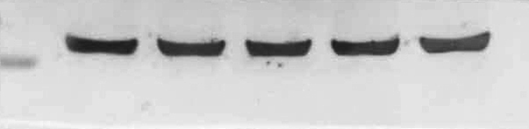


B


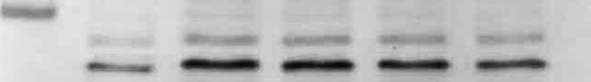

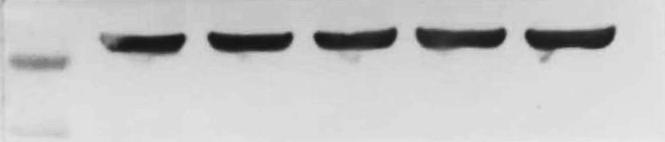


C


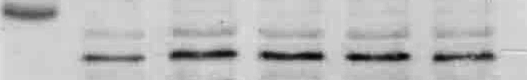

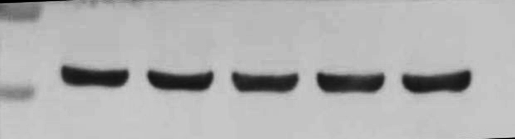


**Supplementary Figure S6.** **The full-length blots/gels images of Figure 4A.**The Figure showed the full-length blots/gels images of Bax and β-actin, in which the Supplementary Figure S6A was the corresponding original uncropped images for Figure 4A in the main manuscript, and other images were parallel test data.

Cty C (14 kd) β-actin (42 kd)

A


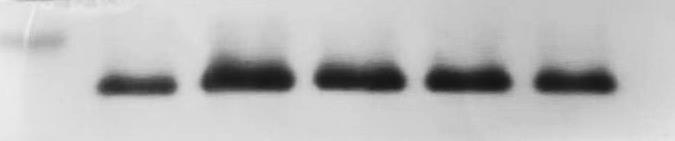

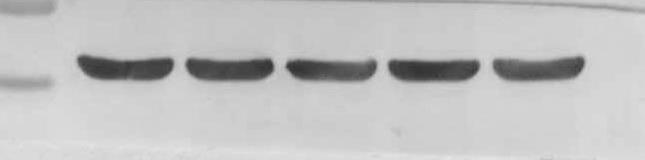


B


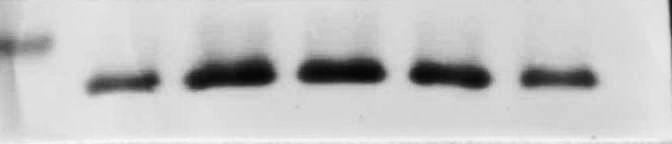

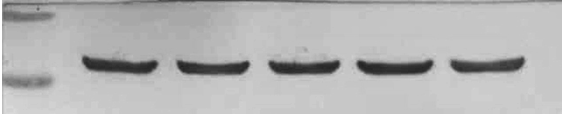


C


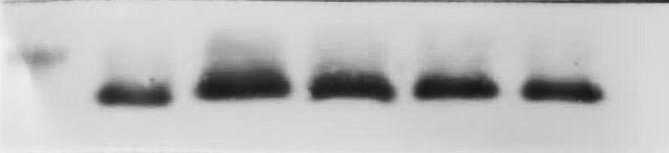

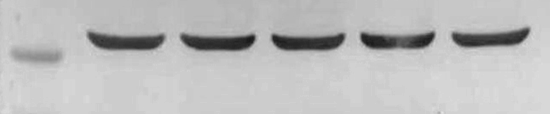


**Supplementary Figure S7.** **The full-length blots/gels images of Figure 4B.**The Figure showed the full-length blots/gels images of Cty C and β-actin, in which the Supplementary Figure S7A was the corresponding original uncropped images for Figure 4B in the main manuscript, and other images were parallel test data.

Bax (23 kd) β-actin (42 kd)

A


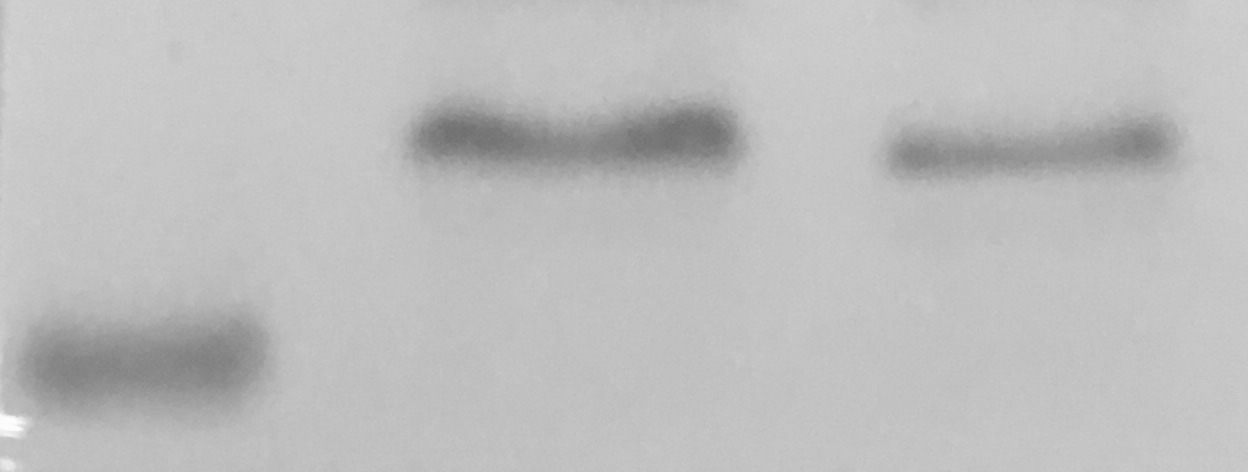

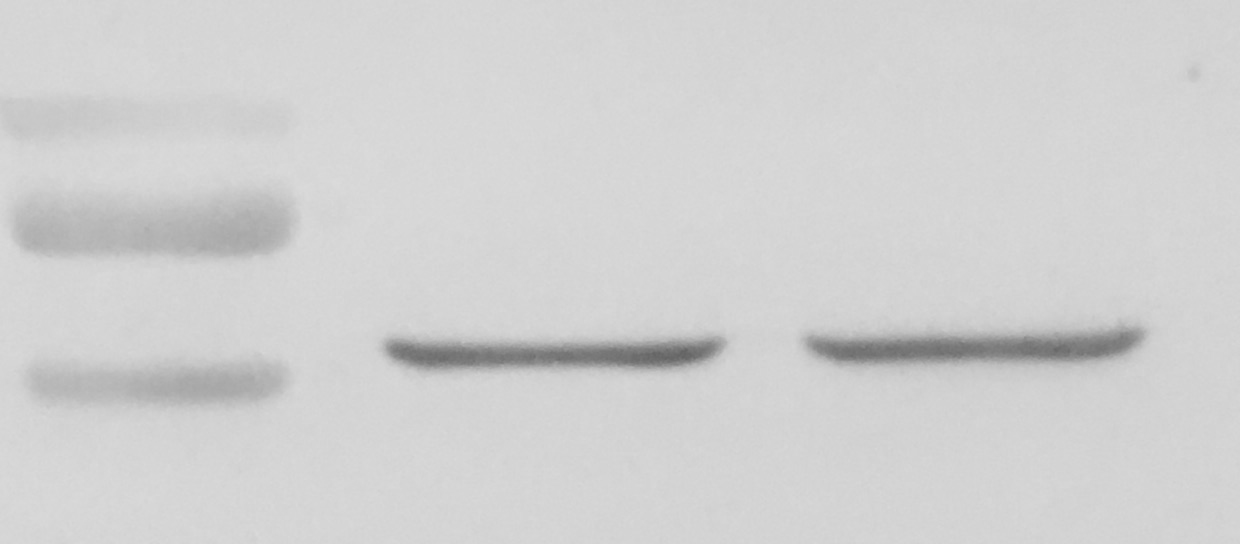


B


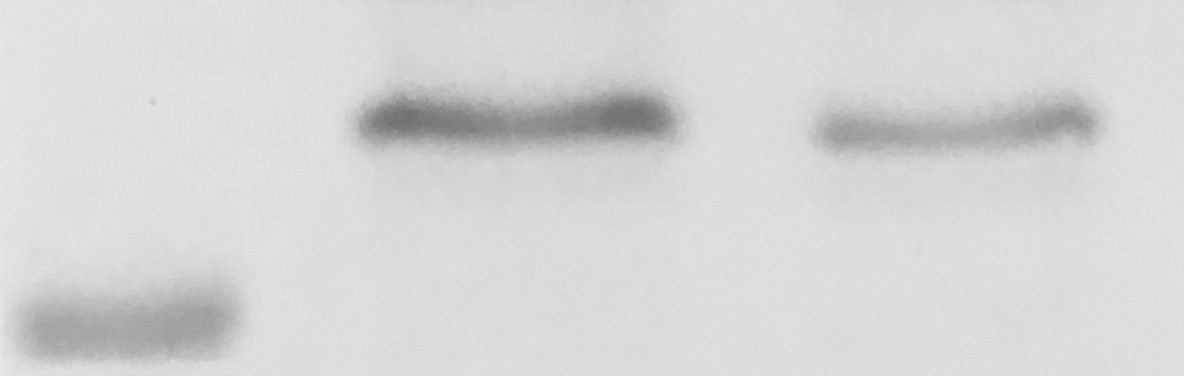

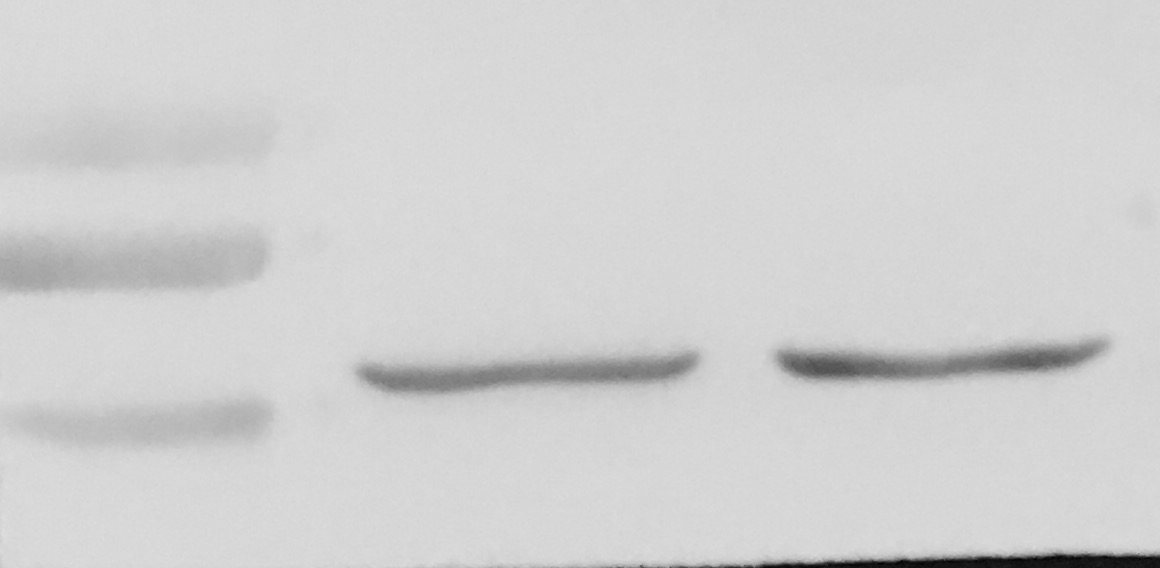


C


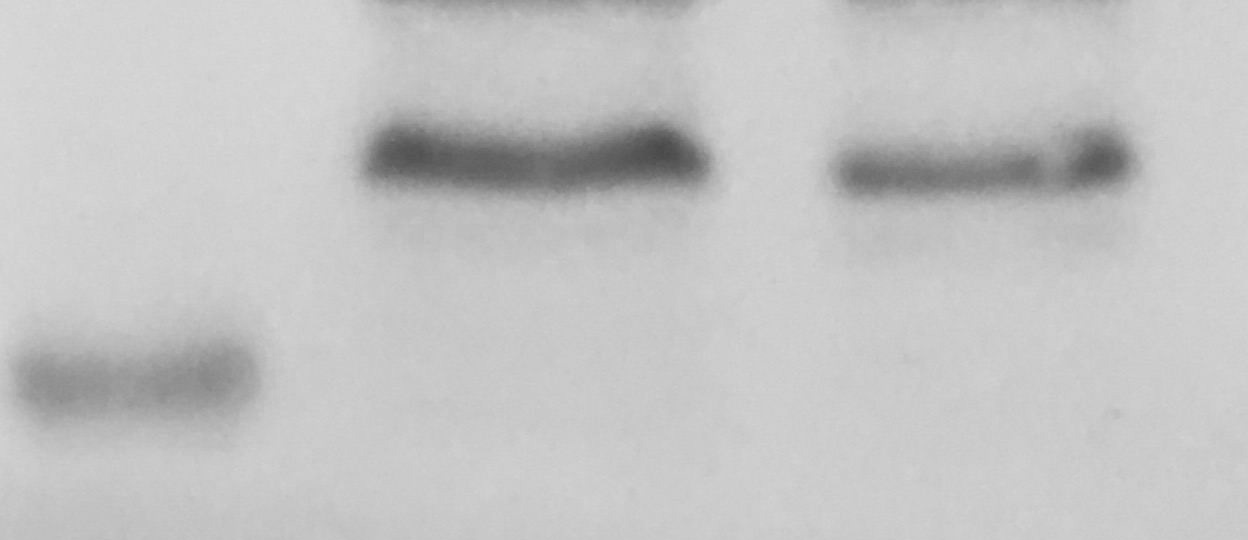

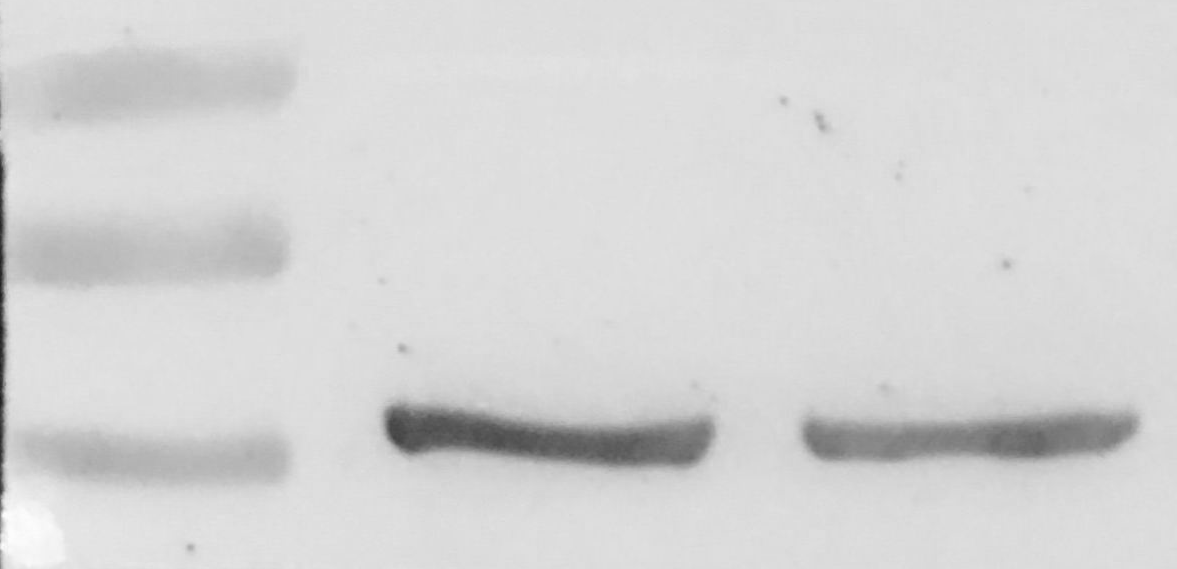


**Supplementary Figure S8.** **The full-length blots/gels images of Supplementary Figure S2A.**The Figure showed the full-length blots/gels images of Bax and β-actin, in which the Supplementary Figure S8A was the corresponding original uncropped images for Supplementary Figure S2A in the main manuscript, and other images were parallel test data.

p-AKT (60 kd) AKT (60 kd)

(Ser473)

A


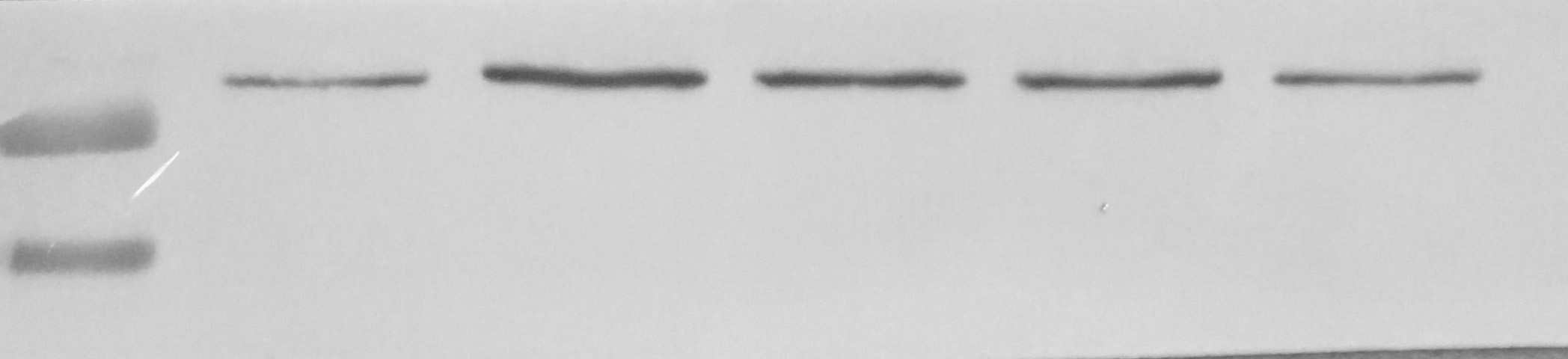

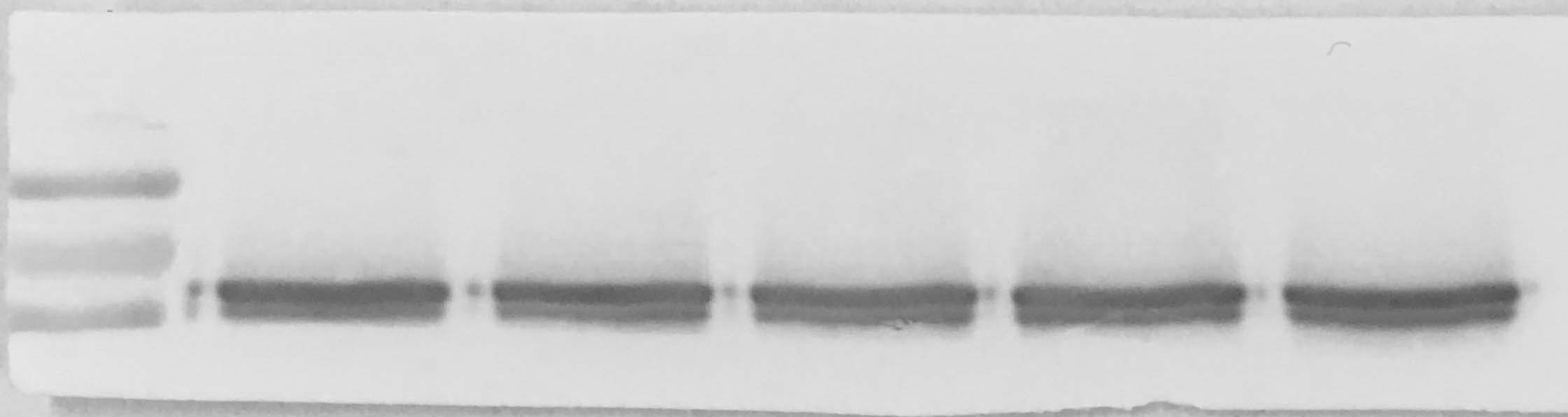


B


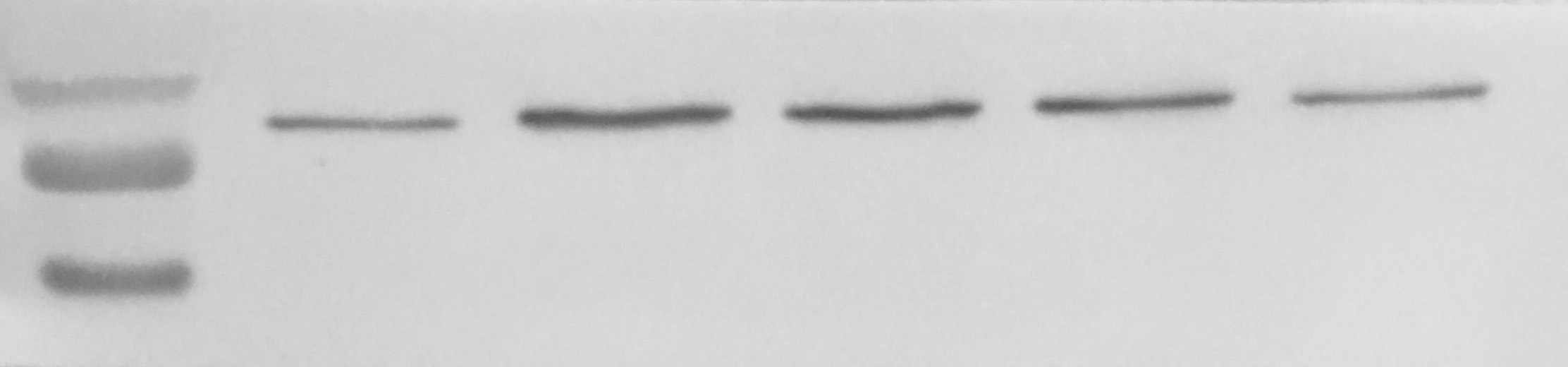

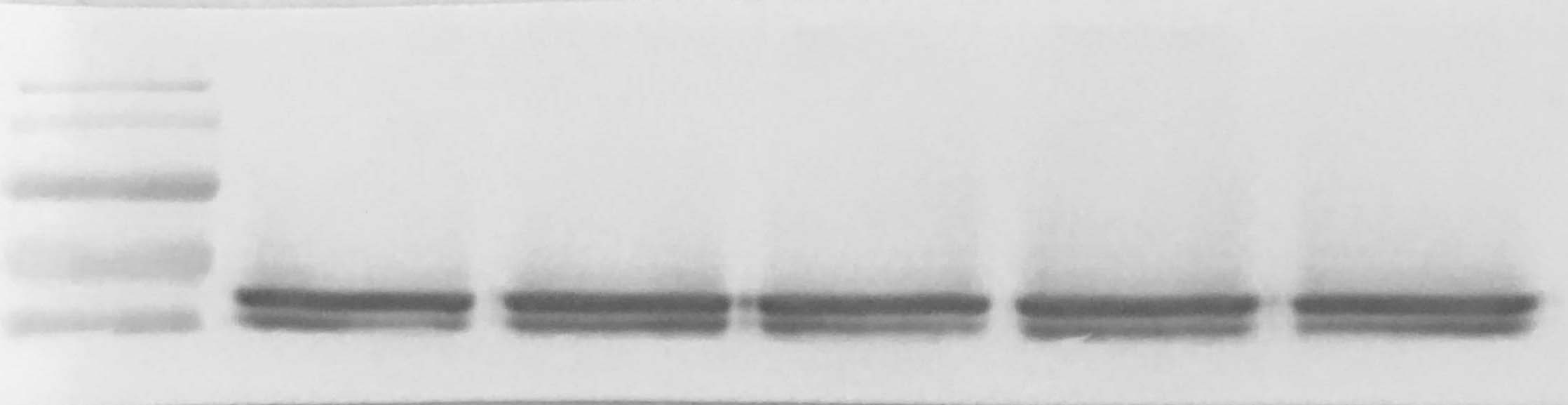


C


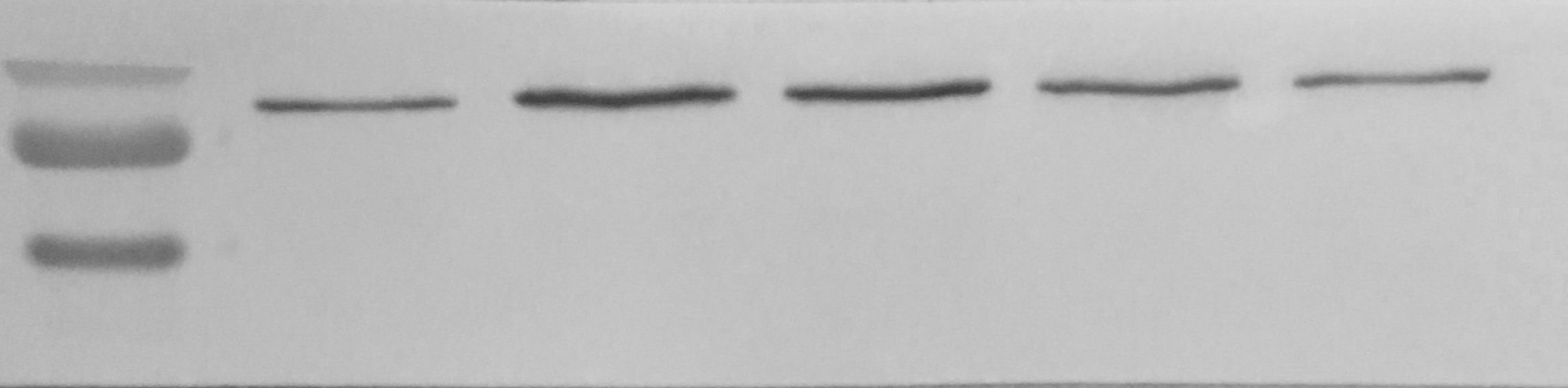

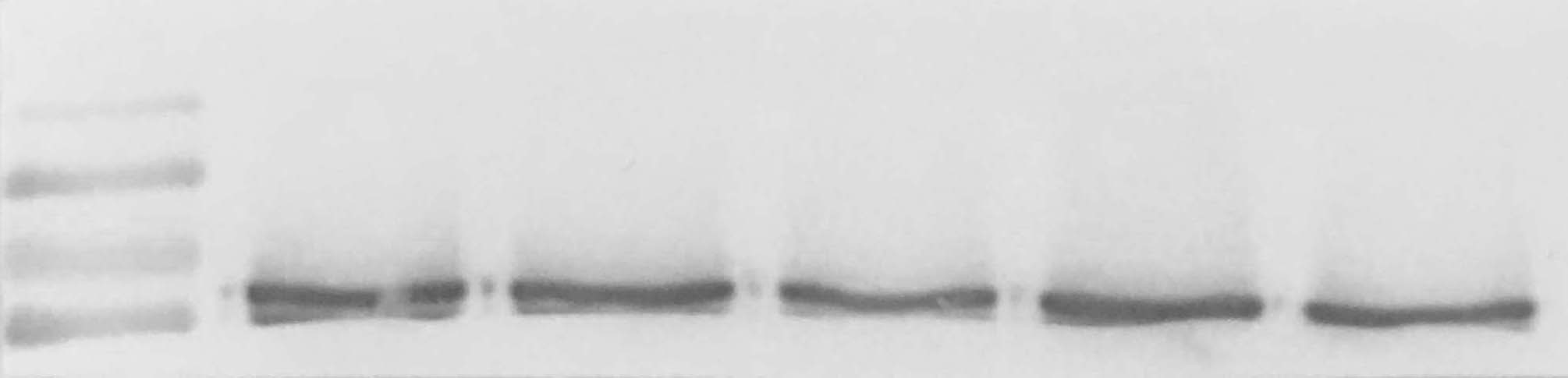


**Supplementary Figure S9.** **The full-length blots/gels images of Supplementary Figure S3A.**The Figure showed the full-length blots/gels images of p-AKT and AKT, in which the Supplementary Figure S9A was the corresponding original uncropped images for Supplementary Figure S3A in the main manuscript, and other images were parallel test data.

p-p70S6K (70 kd) β-actin (42 kd)

(Thr389)

A


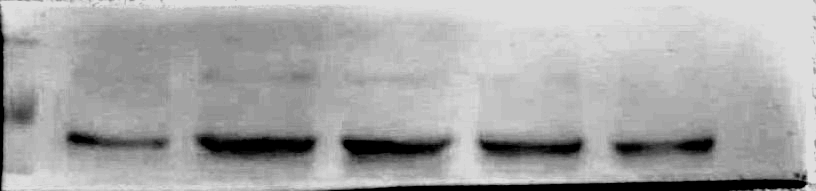

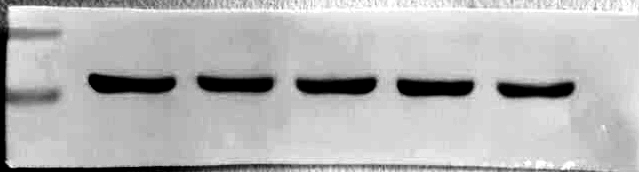


B


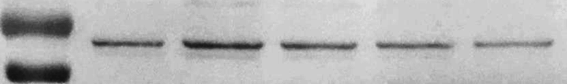

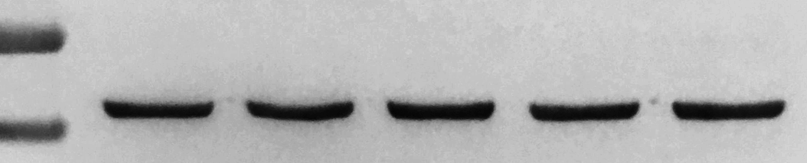


C


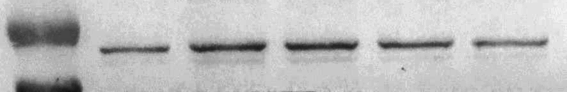

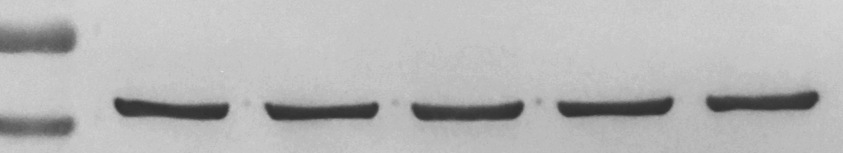


**Supplementary Figure S10.** **The full-length blots/gels images of Supplementary Figure S3B.**The Figure showed the full-length blots/gels images of p-p70S6K and β-actin, in which the Supplementary Figure S10A was the corresponding original uncropped images for Supplementary Figure S3B in the main manuscript, and other images were parallel test data.
